# A population-based nationwide dataset concerning the COVID-19 pandemic and serious psychological consequences in Bangladesh

**DOI:** 10.1016/j.dib.2020.106621

**Published:** 2020-12-05

**Authors:** Amir H. Pakpour, Firoj Al Mamun, Ismail Hosen, Mark D. Griffiths, Mohammed A. Mamun

**Affiliations:** a Social Determinants of Health Research Center, Research Institute for Prevention of Non-Communicable Diseases, Qazvin University of Medical Sciences, Qazvin, Iran; b Department of Nursing, School of Health and Welfare, Jönköping University, Jönköping, Sweden; c Centre for Health Innovation, Networking, Training, Action and Research - Bangladesh, Dhaka, Bangladesh; d Department of Public Health and Informatics, Jahangirnagar University, Savar, Dhaka, Bangladesh; e International Gaming Research Unit, Psychology Department, Nottingham Trent University, Nottingham, United Kingdom

**Keywords:** COVID-19, Knowledge, Behavior, Mental health, Insomnia, Suicidal behavior, Bangladesh

## Abstract

This paper presents the dataset concerning knowledge, preventive behavior, psychological consequences, and suicidal behavior regarding the COVID-19 pandemic in Bangladesh. Data were collected through an online based cross-sectional survey between April 1 and April 10 in 64 districts at the early stage of the COVID-19 pandemic in Bangladesh. A total of 10,067 participants’ data were recruited for analysis. The survey contained items concerning (i) socio-demographic information, (ii) knowledge concerning COVID-19, (iii) behavior towards COVID-19, (iv) lockdown and economic issues, (v) assessment of fear of COVID-19, (vi) assessment of insomnia, (vii) assessment of depression, and (viii) assessment of suicidal ideation. Data were analyzed utilizing SPSS (version 22) and are represented as frequencies and percentages based on responses to the whole survey. Given that the data were collected across the whole nation, government authorities and healthcare policymakers can use the data to develop various models and/or policies regarding preventive strategies and help raise awareness through health education towards COVID-19.

## Specifications Table

**Table:** 

Subject	Infectious diseases and public health
Specific subject area	Health behaviours and psychology
Type of data	Table
How data were acquired	Data were collected utilizing an online survey (i.e., *Google Forms* web-link). A copy of the survey is included as Supplementary File.
Data format	Raw, analysed
Parameters for data collection	The target population were individuals in the 64 districts of Bangladesh. Socio-demographic information, COVID-19 knowledge-related questions, COVID-19 behavior-related questions, Bangla Fear of COVID-19 Scale, Bangla Insomnia Severity Index, Bangla Patient Health Questionnaire, and COVID-19-related suicidal behavior were assessed in the survey*.*
Description of data collection	Non-random convenience sampling using an online data collection platform was used to collect 10,067 participants’ data from a convenience sample from all 64 districts in Bangladesh. The surveys were accessed and completed via social media platform (i.e., *Facebook, WhatsApp, Twitter, Snapchat,* etc.), email, and via other online communicable means.
Data source location	The data were collected by the Department of Public Health and Informatics, Jahangirnagar University, and the Centre for Health Innovation, Networking, Training, Action and Research – Bangladesh (CHINTA Research Bangladesh; which was formally known as the Undergraduate Research Organization), Dhaka, Bangladesh.
Data accessibility	Repository name: Harvard Dataverse Data identification number: doi: https://doi.org/10.7910/DVN/YKH9C1 Direct URL to data: https://dataverse.harvard.edu/dataset.xhtml?persistentId=doi:10.7910/DVN/YKH9C1

## Value of the data

• This dataset is useful because it comprises data from a largescale nationwide study concerning (i) socio-demographics, (ii) COVID-19-related knowledge, (iii) COVID-19-related behavior practices, (iv) lockdown and economic issues, (v) fear of COVID-19, (vi) depression, (vii) sleep patterns and insomnia, and (viii) suicidal ideation.
• Government departments along with non-government organizations can use the dataset for facilitating public policy in relation to COVID-19.
• Screening for suicide and depression can be applied in those regions which are badly affected during the COVOD-19 pandemic.
• These data can be used to make comparisons with the mental health states of populations in other countries (including suicidal ideation).
• To reduce panic and related mental health consequences due to COVID-19, these data can be a major resource for helping developing evidence-based intervention and prevention programs.
  Further analysis of the dataset can be used to aid new methods and/or models to aid good mental health among Bangladeshi people during the COVID-19 pandemic.

## 2 Data Description

As the COVID-19 pandemic has spread out throughout the world, many Bangladeshi communities have been negatively impacted by COVID-19. In Bangladesh, during the early stage of COVID-19 pandemic, an online-based survey was conducted which collected data assessing the level of COVID-19 knowledge, attitudes, and practice among the Bangladeshi general population. The final dataset comprised a total of 10,067 participants. The dataset comprises (i) socio-demographic characteristics (e.g., gender, age group, educational status, occupational status, data discipline, residence area, marital status, comorbidities, current health condition, smoking status, alcohol-drinking status, frequency of social media use, etc.) (Table 1); (ii) sources from where participants get information regarding COVID-19 (e.g., social media, *YouTube*, newspaper, television, health-related website, and other sources) (Table 1); (iii); participants’ knowledge concerning COVID-19 (Table 2); (iv) participants’ behavior in preventing COVID-19 (Table 3); (v) lockdown-related questions (Table 4); (vi) assessment of fear of COVID-19 among participants (Table 5); (vii) assessment of severity of insomnia among participants (Table 6); (viii) assessment of depression among participants (Table 7); and (ix) suicidal ideation in relation to COVID-19 among participants (Table 7). Detailed information concerning all of the variables are shown in Tables 1–8. A copy of the complete survey can be accessed as a Supplementary File.

**Table 1:** Distribution of responses in relation to socio-demographic variables.

Socio – demographics			Frequency	Percentages
**Age group; Mean ± SD = 26.94** **±** **9.63 years**				
10–19 years			685	6.8
20–29 years			7175	71.3
30–39 years			1221	12.1
40–49 years			410	4.1
50–59 years			371	3.7
60 years and above (elderly)			196	1.9
**Gender**				
Male			5650	56.1
Female			4402	43.7
**Educational status**				
No formal education			197	2.0
Primary level (up to 5)			169	1.7
Secondary level (6 to 10)			427	4.2
Higher secondary level (11–12)			1139	11.3
Tertiary level			8135	80.8
**Occupational status**				
Unemployed			361	3.6
Day-laborer			79	0.8
Farmer			73	07
Businessman			492	4.9
Student			5878	58.4
Government employee			561	5.6
Private employee			1381	13.7
Retired			92	0.9
Housewife			713	7.1
Others			437	4.3
**Data discipline**				
Pure science			833	8.3
Medical or allied health sciences			2014	20.0
Arts or social science			1257	12.5
Engineering			1264	12.6
Business studies			1052	10.4
Others			1232	12.2
**Divisional residence**				
Barisal			207	2.1
Chittagong			2048	23.9
Dhaka			4292	42.6
Khulna			1045	10.4
Mymensingh			258	2.6
Rajshahi			946	9.4
Sylhet			333	3.3
**Administrative residence**				
Village			2336	23.2
Upazilla town			1359	13.5
District level town			2334	23.2
Divisional city			4038	40.1
**Marital status**				
Unmarried			7081	70.3
Married			2839	28.2
Divorced			40	0.4
Widower			22	0.2
Widow			62	0.6
Others			23	0.2
**Smoking status**				
Yes			1486	14.8
No			8581	85.2
**Alcohol drinking status**				
Yes			267	2.7
No			9800	97.3
**Current health status**				
Very good			6909	68.6
Acceptable			2811	27.9
Poor			312	3.1
Very poor			35	0.3
**Current diseases**				
Diabetics		Yes	399	4.0
		No	2078	20.6
High blood pressure		Yes	585	5.8
		No	1892	18.8
Asthma/respiratory problem		Yes	752	7.5
		No	1725	17.1
Heart disease		Yes	126	1.3
		No	2351	23.4
Kidney problem		Yes	83	0.8
		No	2394	23.8
Cancer		Yes	10	0.1
		No	2467	24.5
Other diseases not listed		Yes	1114	11.1
		No	1363	13.5
**Taking naps during the day; Mean ± SD = 1.94** **±** **0.74**				
Very likely			3042	30.2
Somewhat likely			4563	45.3
Not likely			2462	24.5

**From Dhaka after March 17, 2020**				
Yes			1294	12.9
No			7671	76.2
**From COVID-19 infected country after January 2020**				
Yes			256	2.5
No			9811	97.5
**Social media user**				
Yes			9152	90.9
No			915	9.1
**Frequency of social media use**				
More than 4 days a week			292	2.9
2 or 3 days a week			318	3.2
Everyday			4082	40.5
Several times a day			4451	44.2
**Sources of information regarding COVID-19**				
Social media	Yes		8277	82.2
	No		1790	17.8
YouTube	Yes		4365	43.4
	No		5702	56.6
Newspaper	Yes		4933	49.0
	No		5134	51.0
Television	Yes		7306	72.6
	No		2761	27.4
Health-related websites	Yes		4498	44.7
	No		5569	55.3
Other sources	Yes		1948	19.4
	No		8119	80.6

**Table 2:** Distribution of responses in relation to COVID-19 knowledge-related variables.

Knowledge related questions				Frequency	Percentages
Spreading	Can be spread from infected individuals cough or exhalation		Yes	9905	98.4
			No	162	1.6
	Can be spread from infected individuals by touch		Yes	8732	86.7
			No	1335	13.3
	Can be spread from wild animals		Yes	2857	28.4
			No	7210	71.6
	Can be spread form infected individuals faces		Yes	2350	23.3
			No	7717	76.7
	Can be spread from companion animals or pets such as cats and dogs		Yes	3141	31.2
			No	6926	68.8
	Can be spread through parcels from infected countries		Yes	2009	20.0
			No	8058	80.0
Symptoms	Has an incubation period ranging from 2 to 14 days		Yes	9416	93.5
			No	651	6.5
	Individuals may not develop any symptoms		Yes	6525	64.8
			No	3542	35.2
	The most common symptoms are fever, tiredness, and dry cough		Yes	8500	84.4
			No	1567	15.6
	Individuals may develop respiratory problems		Yes	8478	84.2
			No	1589	15.8
	Some individuals may have aches and pains, nasal congestion, runny nose, sore throat, or diarrhea.		Yes	7525	74.7
			No	2542	25.3
	Individuals with comorbidities are more likely to develop serious illness (e.g., organ failure)		Yes	6332	62.9
			No	3735	37.1
Preventive measures	Washing hands regularly for 20 s		Yes	9801	97.4
			No	266	2.6
	Avoid touching eyes, nose, and mouth		Yes	9602	95.4
			No	465	4.6
	Wearing masks is mandatory		Yes	8897	88.4
			No	1170	11.6
	Avoiding close contact from the infected individuals		Yes	9455	93.9
			No	612	6.1
	Maintain at least one-meter (three feet) distance between yourself and anyone who is coughing or sneezing.		Yes	9076	90.2
			No	991	9.8
	Quarantine at home if you feel unwell and isolate the infected individual		Yes	9244	91.8
			No	823	8.2
Treatments	Taking pills such as paracetamol		Yes	3831	38.1
			No	6236	61.9
	To date, there is no vaccine and no specific antiviral medicine to prevent or treat COVID-2019		Yes	8919	88.6
			No	1148	11.4

**Table 3:** Distribution of responses related to COVID-19 preventive behaviors.

Preventive behavior related questions		Frequency	Percentages
Cleaning hands with an alcohol-based hand rub or wash them with soap and water	Never	87	0.9
	Seldom	196	1.9
	Sometimes	965	9.6
	Often	3059	30.4
	Almost always	5760	57.2
Practicing respiratory hygiene (covering mouth and nose with bent elbow or tissue when coughing or sneezing).	Never	307	3.0
	Seldom	318	3.2
	Sometimes	983	9.8
	Often	2036	20.2
	Almost always	6423	63.8
Maintaining at least one-meter (three feet) distance from anyone who is coughing or sneezing	Never	463	4.6
	Seldom	1157	11.5
	Sometimes	2083	20.7
	Often	3322	33.0
	Almost always	3042	30.2
Staying at home if feeling unwell	Never	222	2.2
	Seldom	365	3.6
	Sometimes	856	8.5
	Often	2076	20.6
	Almost always	6176	61.3
Self-isolating or staying at home for seven days	Not a single day	781	7.8
	1 day	93	0.9
	2 days	140	1.4
	3 days	256	2.5
	4 days	320	3.2
	5 days	488	4.8
	6 days	491	4.9
	7 days	7498	74.5
Going outside for 15 min or more in the past 7 days	Not a single day	4920	48.9
	1 day	1279	12.7
	2 days	1155	11.5
	3 days	780	7.7
	4 days	409	4.1
	5 days	332	3.3
	6 days	168	1.7
	7 days	1024	10.2
Had face-to-face contact with another individual for 15 min or more in past seven days	Not a single day	5088	50.5
	1 day	1820	18.1
	2 days	952	9.5
	3 days	669	6.6
	4 days	346	3.4
	5 days	265	2.6
	6 days	131	1.3
	7 days	796	7.9

**Table 4:** Distribution of responses related to lockdown-related variables.

Lockdown-related question			Frequency	Percentages
Problems faced during lockdown	Feeling uncomfortable	Yes	6391	63.5
		No	3676	36.5
	Cannot buy necessary things	Yes	4262	42.3
		No	5805	57.7
	Unable to maintain usual daily routine like before	Yes	6066	60.3
		No	4001	39.7
	Unable to engage in daily physical exercise	Yes	3231	32.1
		No	6836	67.9
	Afraid of going out to sunbathe (e.g., open place, corridor, terrace)	Yes	1829	18.2
		No	8238	81.8
	Unable to play in the field	Yes	1902	18.9
		No	8165	81.1
	Unable to concentrate on household activities	Yes	2780	27.6
		No	7287	72.4
	Facing other problems not listed here	Yes	3689	36.6
		No	6378	63.4
Having enough food supply	Agree		2001	19.9
	Disagree		3855	38.3
	Undecided		4211	41.8
Experiencing panic due to economic recession	Agree		8814	87.6
	Disagree		624	6.2
	Undecided		629	6.2
Having economic hardship	Agree		4283	42.5
	Disagree		1373	13.6
	Undecided		2230	22.2

**Table 5:** Distribution of responses on the fear of COVID-19 scale.

Fear of COVID-19 Scale (FCV-19S)		Frequency	Percentages
I am most afraid of Coronavirus-19	Strongly disagree	558	5.5
	Disagree	1083	10.8
	Neither agree nor disagree	1881	18.7
	Agree	4898	48.7
	Strongly agree	1647	16.4
It makes me uncomfortable to think about Coronavirus-19	Strongly disagree	611	6.1
	Disagree	1434	14.2
	Neither agree nor disagree	1584	15.7
	Agree	5125	50.9
	Strongly agree	1313	13.0
My hands become clammy when I think about Coronavirus-19	Strongly disagree	1998	19.8
	Disagree	3820	37.9
	Neither agree nor disagree	2018	20.0
	Agree	1764	17.5
	Strongly agree	467	4.6
I am afraid of losing my life because of Coronavirus-19	Strongly disagree	1516	15.1
	Disagree	2681	26.6
	Neither agree nor disagree	1757	17.5
	Agree	3336	33.1
	Strongly agree	777	7.7
When watching news and stories about Coronavirus-19 on social media, I become nervous or anxious.	Strongly disagree	738	7.3
	Disagree	1312	13.0
	Neither agree nor disagree	1156	11.5
	Agree	5769	57.3
	Strongly agree	1092	10.8
I cannot sleep because I'm worrying about getting Coronavirus-19	Strongly disagree	2074	20.6
	Disagree	4287	42.6
	Neither agree nor disagree	1548	15.4
	Agree	1751	17.4
	Strongly agree	407	4.0
My heart races or palpitates when I think about getting Coronavirus-19	Strongly disagree	1509	15.0
	Disagree	3216	31.9
	Neither agree nor disagree	1368	13.6
	Agree	3131	31.1
	Strongly agree	843	8.4

**Table 6:** Distribution of responses on the Insomnia Severity Index.

Insomnia Severity Index (ISI)		Frequency	Percentages
Difficulty falling asleep	None	3548	35.2
	Mild	1945	19.3
	Moderate	2404	23.9
	Severe	1247	12.4
	Very severe	923	9.2
Difficulty staying asleep	None	4441	44.4
	Mild	–	–
	Moderate	4370	43.4
	Severe	948	9.4
	Very severe	308	3.1
Problems waking up too early	None	5607	55.7
	Mild	1425	14.2
	Moderate	1968	19.5
	Severe	765	7.6
	Very severe	302	3.0
How SATISFIED/DISSATISFIED are you with your CURRENT sleep pattern?	Very satisfied	1759	17.5
	Satisfied	3622	36.0
	Moderately satisfied	2819	28.0
	Dissatisfied	1294	12.9
	Very dissatisfied	573	5.7
How NOTICEABLE to others do you think your sleep problem is in terms of impairing the quality of your life?	Not at all noticeable	5769	57.3
	A little	1533	15.2
	Somewhat	1980	19.7
	Much	472	4.7
	Very much noticeable	313	3.1
How WORRIED/DISTRESSED are you about your current sleep problem?	Not at all worried	5300	52.6
	A little	1989	19.8
	Somewhat	1620	16.1
	Much	803	8.0
	Very much worried	355	3.5
To what extent do you consider your sleep problem to INTERFERE with your daily functioning (e.g., daytime fatigue, mood, ability to function at work/daily chores, concentration, memory, mood, etc.) CURRENTLY?	Not at all interfering	4443	44.1
	A little	1929	19.2
	Somewhat	2387	23.7
	Much	748	7.4
	Very much interfering	560	5.6

**Table 7:** Distribution of responses on the Patient Health Questionnaire.

Patient Health Questionnaire (PHQ-9)		Frequency	Percentages
Little interest or pleasure in doing things	Not at all	2175	21.6
	Several days	5087	50.5
	More than half days	1623	16.1
	Nearly everyday	1182	11.7
Feeling down, depressed or hopeless	Not at all	2445	24.3
	Several days	5083	50.5
	More than half days	1529	15.2
	Nearly everyday	1010	10.0
Trouble falling or staying asleep, or sleeping too much	Not at all	3400	33.8
	Several days	3970	39.4
	More than half days	1560	15.5
	Nearly everyday	1137	11.3
Feeling tired or having little energy	Not at all	3470	34.5
	Several days	4533	45.0
	More than half days	1320	13.1
	Nearly everyday	744	7.4
Poor appetite or overheating	Not at all	4979	49.5
	Several days	3444	34.2
	More than half days	1046	10.4
	Nearly everyday	598	5.9
Feeling bad about yourself-or that you are a failure or have let yourself or your family down	Not at all	5903	58.6
	Several days	2739	27.2
	More than half days	739	7.3
	Nearly everyday	686	6.8
Trouble concentrating on things, such as reading the newspaper or watching television	Not at all	3222	32.0
	Several days	–	–
	More than half days	5632	55.9
	Nearly everyday	1213	12.0
Moving or speaking so slowly that other people could have noticed. Or the opposite-being so fidgety or restless that you have been moving around a lot more than usual	Not at all	6697	66.5
	Several days	2421	24.0
	More than half days	607	6.0
	Nearly everyday	342	3.4
Thoughts that you would be better off dead, or of hurting yourself	Not at all	8290	82.3
	Several days	1228	12.2
	More than half days	284	2.8
	Nearly everyday	265	2.6

**Table 8:** Distribution of responses related to suicidal behavior.

Suicide-related question		Frequency	Percentages
“Do you think about committing suicide, and are these thoughts persistent and related to COVID-19 issues?”	Yes	506	5.0
	No	9581	95.0

## 3 Experimental Design, Materials and Methods

Cross-sectional data collection was carried out among 64 districts of Bangladesh between April 1 and 10 (2020). In each district, three or four research assistants (approximately 250 in total) were utilized to facilitate the completion of an online survey form via social media platforms among individuals living in those districts (approximately 250 RAs). A total of 10,067 participants out of approximately 11,000 were eligible. The inclusion criteria were (i) being Bangladeshi, (ii) residing in Bangladesh, and (iii) being aged over 10 years.

The survey comprised socio-demographic information including age, gender, educational status, occupational status, current place of residence, marital status, current cigarette smoking behavior (yes/no), current alcohol-drinking behavior (yes/no), and frequency of social media use. Current health status was assessed using a single question (i.e., “Are you suffering from any of the following health-related issues?”) with seven response choices (i.e., diabetes, high blood pressure, asthma/respiratory problem, heart disease, kidney problems, cancer, and any other health conditions not listed) where each positive response was scored as one point.

COVID-19 knowledge was assessed based on questions relating to: (i) spread of infection (six true/false statements; e.g. *‘COVID-19 can spread by touching others’*), (ii) symptoms (six true/false statements; e.g., *‘The most common symptoms of COVID-19 are fever, tiredness, and dry cough’*), (iii) prevention behaviors (six true/false statements; e.g*., ‘Washing hands regularly for 20* *s’)*, and (iv) treatment (two statements; e.g., *‘Taking pills like antibiotics when you have fever’*). To create a total COVID-19 knowledge score, each correct answer scored one point and incorrect answers scored zero. All responses are summed to calculate a total score ranging from 0 to 20 where higher scores reflected better knowledge concerning COVID-19. There is no recoding of any items in calculating the total score [1].

COVID-19 preventive behavior was assessed based on four items (e.g., *“How often do you clean your hands with an alcohol-based hand rub or wash them with soap and water?”*) responded to on a five-point Likert scale from 1 (*never*) to 5 (*almost always*). All items are summed to calculate a total score ranging from 4 to 20, with higher scores reflecting higher performing COVID-19 preventive behaviors.

Fear of COVID-19 was assessed using the Bangla Fear of COVID-19 Scale which comprises seven items (e.g., *‘I am afraid of losing my life because of Coronavirus-19′*) responded to on a five-point Likert scale from 1 (*strongly disagree*) to 5 (*strongly agree*). All items are summed to calculate a total score ranging from 7 to 35, with higher scores indicating higher fear of COVID-19. There is no recoding of any items in calculating the total score [1,2].

Insomnia was assessed using the Bangla Insomnia Severity Index which comprises seven item (e.g., *“How satisfied/dissatisfied are you with your current sleep pattern?”*) responded to on a five-point Likert scale from 0 (*very satisfied*) to 4 (*very dissatisfied*). All items are summed up to calculate a total score ranging from 0 to 28, with higher scores indicating higher insomnia symptomology. There is no recoding of any items in calculating the total score [3].

Depression was assessed using the Bangla Patient Health Questionnaire which comprises nine items (e.g., *“Little or interest or pleasure in doing things”*) responded to on a five-point Likert scale from 0 (not at all) to 3 (nearly every day). All items are summed to calculate a total score ranging from 0 to 27, with higher scores indicating higher levels of depression. There is no recoding of any items in calculating the total score [4,5].

COVID-19-related suicidal behavior was assessed using a binary (yes/no) response to a single question (*“Do you think about committing suicide, and are these thoughts persistent and related to COVID-19 issues?”*) which was used in previous Bangladeshi studies [5,6]. Data were analyzed using the Statistical Packages for Social Science (SPSS) version 23.0, AMOS version 23.0 and ArcGIS 10.5 for analysis. Frequency and percentages were calculated.

## Ethics Statement

In collecting the data, the 1975 Helsinki declaration and ethical permission to collect the data was granted from Biosafety, Biosecurity, and Ethical Committee of Jahangirnagar University, Bangladesh (BBEC, JU/M 2O20/COVlD-l9/(9)2) and the Institute of Allergy and Clinical Immunology of Bangladesh ethics board, Bangladesh (IRBIACIB/CEC/03202005). Additionally, written informed consent was provided by all participants prior to starting the survey. They were informed about the purpose and nature of the data and they had the right to withdraw their data if they wanted to. For participants under 18 years, parental consent was taken and all the participants were assured about the confidentiality of their data.

## CRediT Author Statement

Amir H. Pakpour: Conceptualization, Investigation, Writing original draft and Analyses; Firoj Al Mamun: Conceptualization and Investigation; Ismail Hosen Conceptualization and Investigation; Mark D. Griffiths: Writing, Review and Editing; Mohammed A. Mamun: Conceptualization, Investigation, Writing original draft, Analyses and validation.

## Declaration of Competing Interest

The authors declare that they have no known competing financial interests or personal relationships that could have appeared to influence the work reported in this paper.

## Appendix Supplementary materials

Image, application 1

## References

[1] Sakib N., Bhuiyan A.K.M.I., Hossain S., Al Mamun F., Hosen I., Abdullah A.H. (2020). Psychometric validation of the Bangla fear of COVID-19 scale: confirmatory factor analysis and Rasch analysis. Int. J. Ment. Health Addict..

[2] Ahorsu D.K., Lin C.-.Y., Imani V., Saffari M., Griffiths M.D., Pakpour A.H (2020). The fear of COVID-19 scale: development and initial validation. Int. J. Ment. Health Addict..

[3] Mamun M.A., Pakpour A.H.,. Psychometric validation of Bangla insomnia severity index: confirmatory factor analysis and Rasch analysis. Under review 2020.

[4] Mamun M.A., Huq N., Papia Z.F., Tasfina S., Gozal D (2019). Prevalence of depression among Bangladeshi village women subsequent to a natural disaster: a pilot study. Psychiatry Res..

[5] Jahan S., Araf K., Gozal D., Griffiths M.D., Mamun M.A (2020). Depression and suicidal behaviors among Bangladeshi mothers of children with autism spectrum disorder: a comparative study. Asian J. Psychiatr..

[6] Mamun M.A., Akter T., Zohra F., Sakib N., Bhuiyan A.K.M.I., Banik P.C. (2020). Prevalence and risk factors of COVID-19 suicidal behavior in Bangladeshi population: are healthcare professionals at greater risk?. Heliyon.

