# Supplementary material for: A population-based nationwide dataset concerning the COVID-19 pandemic and serious psychological consequences in Bangladesh
Source: Data Brief. 2020 Dec 5;33:106621. doi: 10.1016/j.dib.2020.106621 (PMC7736911; doi:10.1016/j.dib.2020.106621)
Supplement: Supplementary file 1 [file mmc1.docx]

***Knowledge and behaviors towards COVID-19 and its psychological impact among Bangladeshi people: A nationwide survey***

***Consent***

**Do you want to participate in this survey?**

1. Yes
2. No

***Socio-demographics (SD)***

**SD_1: Your age in years (write in number, e.g., 20)**

…………………………..

**SD_2: Gender**

1. Male
2. Female
3. Others

**SD_3: Educational qualification**

1. No formal education
2. Primary level (Up to class 5)
3. Secondary school level (class 6 to 10)
4. Higher secondary level (class 11 to 12)
5. Tertiary education

**SD_4: Occupation**

1. Unemployed
2. Day-laborer
3. Farmer
4. Business
5. Student
6. Government employee
7. Private employee
8. Retired
9. Housewife
10. Others

**SD_5: If you currently a *student*, what is your discipline?**

1. Pure science
2. Medical or allied health science
3. Arts or social science
4. Engineering
5. Business studies
6. Others

**SD_6: Current place of residence (e.g., Dhaka district)**

…………………………..

**SD_7: Which type of administrative region are you living in?**

1. Village
2. Upazilla town
3. District-level town
4. Divisional city

**SD_8: Marital status**

1. Unmarried
2. Married
3. Divorced
4. Widower
5. Widow
6. Others

**SD_9: Do you smoke?**

1. Yes
2. No

**SD_10: Do you consume alcohol?**

1. Yes
2. No

**SD_11: What do you think of your current health condition?**

1. Very good
2. Good
3. Acceptable
4. Poor
5. Very poor

**SD_12: Are you suffering from any of the following health-related issues? (You can choose more than one answer) (If you do not have any, please proceed to the next question)**

1. Diabetics
2. High blood pressure
3. Asthma/ Respiratory problem
4. Heart disease
5. Kidney problem
6. Cancer
7. Any other not listed

**SD_13: What time do you generally go to sleep at night? (e.g., 11:00 pm)**

……………………..

**SD_14: What time do you wake up usually? (e.g., 6:30 am)**

…………………………

**SD_15: Do you take naps during the day?**

1. Very likely
2. Somewhat likely
3. Not likely

**SD_16: If “YES”, how long do you take naps? (Write in minutes, e.g., 30) (If your previous answer is ‘NO’, skip the question)**

………………………………….

**SD_17: If you reside outside Dhaka, is there anyone come to your home from Dhaka after 17th March 2020?**

1. Yes
2. No

**SD_18: Did anyone come to your home from any COVID-19 affected countries after January 2020?**

1. Yes
2. No

**SD_19: Do you use social media like Facebook, Twitter, WhatsApp or others?**

1. Yes
2. No

**SD_20: If you use *social media*, what is the frequency of its use?**

1. More than 4 days a week
2. 2-3 days a week
3. Everyday
4. Several times a day

**SD_21: From where you get information regarding COVID-19 (multiple answers)**

1. Social media (e.g. Facebook, WhatsApp, Twitter, etc.)
2. YouTube
3. Newspaper
4. Television
5. Health-related websites (e.g. DGHealth, WHO, etc.)
6. Other sources not listed here

***Lockdown-related questions (LRQs)***

**LRQ_1: Nobody is allowed to go outside home without any emergency; everything except groceries and pharmacies is closed. Which are the following problems you are facing in this lockdown situation? (You can choose more than one answer)**

1. Feeling uncomfortable
2. Cannot buy other necessary things
3. Unable to maintain the usual daily routine like before
4. Unable to involve in daily physical exercises
5. Afraid of going out (e.g., open place, corridor, terrace) to san-bathe
6. Unable to play in the field
7. Unable to concentrate on household activities
8. Facing other problems, no listed here

**LRQ_2: If this lockdown situation persists more than a month, do you think you will have enough food supply?**

1. Agree
2. Disagree
3. Undecided

**LRQ_3: Are you panicked of any probable economic recession due to this pandemic?**

1. Agree
2. Disagree
3. Undecided

**LRQ_4: If you are a wage earner, do you think you may lose your job and/or face economic hardship in business?**

1. Agree
2. Disagree
3. Undecided

***Knowledge-related questions (KRQs)***

**KRQ_1: Which of the following statements you think ‘TRUE’ in terms of spreading COVID-19? (You can choose more than one answer)**

1. Can spread from the COVID-19 affected persons through coughs or exhales.
2. Can spread from the COVID-19 affected persons by touching others
3. Do not spread from wild animals to humans
4. Do not spread from the feces of the COVID-19 affected persons
5. Do not spread from companion animals or pets such as cats and dogs to humans
6. Do not spread through the parcels coming from COVID-19 affected countries

**KRQ_2: Which of the following symptoms you think ‘TRUE’ in the case of COVID-19? (You can choose more than one answer)**

1. Incubation period for COVID-19 ranging from 2-14 days
2. Some people may not develop any symptoms
3. The most common symptoms of COVID-19 are fever, tiredness, and dry cough.
4. Respiratory problems/ Pneumonia may develop
5. Some patients may have aches and pains, nasal congestion, runny nose, sore throat or diarrhea.
6. Those with underlying medical problems like high blood pressure, heart problems or diabetes, are more likely to develop serious illness (e.g., organ failure)

**KRQ_3: Which of the following preventive measures you think can be taken in the case of COVID-19? (You can choose more than one answer)**

1. Washing hands regularly for 20 seconds
2. Avoid touching eyes, nose and mouth
3. Wearing masks is mandatory
4. Avoiding close contact from the affected persons
5. Maintain at least 1-meter (3 feet) distance between yourself and anyone who is coughing or sneezing.
6. Maintain home quarantine if you feel unwell and isolate the affected person

**KRQ_4: What are the treatments one can follow for COVID-19? (You can choose more than one answer)**

1. Taking antibiotics
2. Taking pills like Paracetamols
3. Taking cow feces and urine, or other herbal medicine
4. Taking any other traditional alternative medicine
5. To date, there is no vaccine and no specific antiviral medicine to prevent or treat COVID-2019.

***Behavior-related questions (BRQs)***

**BRQ_1: How often do you clean your hands with an alcohol-based hand rub or wash them with soap and water?**

1. Never
2. Seldom
3. Sometimes
4. Often
5. Almost always

**BRQ_2: How often do you Practice respiratory hygiene (covering your mouth and nose with your bent elbow or tissue when you cough or sneeze)?**

1. Never
2. Seldom
3. Sometimes
4. Often
5. Almost always

**BRQ_3: How often do you maintain at least 1-meter (3 feet) distance between yourself and anyone who is coughing or sneezing?**

1. Never
2. Seldom
3. Sometimes
4. Often
5. Almost always

**BRQ_4: How often do you stay home if you feel unwell?**

1. Never
2. Seldom
3. Sometimes
4. Often
5. Almost always

**BRQ_5: In the past 7 days, how many days have you been self-isolating or staying at home?**

1. 1 day
2. 2 days
3. 3 days
4. 4 days
5. 5 days
6. 6 days
7. 7 days
8. Not a single day

**BRQ_6: In the past 7 days, how many days have you been outside for 15 minutes or more?**

1. 1 day
2. 2 days
3. 3 days
4. 4 days
5. 5 days
6. 6 days
7. 7 days
8. Did not go out at all

**BRQ_7: In the past 7 days, how many days have you had face-to-face contact with another person for 15 minutes or more?**

1. 1 day
2. 2 days
3. 3 days
4. 4 days
5. 5 days
6. 6 days
7. 7 days
8. Did not go out at all

***Fear of COVID-19 Scale (FCV-19S)***

**Answer to the following statements using a five-item Likert-type scale.**

**Answers included: 1. Strongly disagree 2. Disagree 3. Neither agree nor disagree 4. Agree 5. Strongly agree**

**FCV-19S_1:** I am most afraid of Coronavirus-19

**FCV-19S_2:** It makes me uncomfortable to think about Coronavirus-19

**FCV-19S_3:** My hands become clammy when I think about Coronavirus-19

**FCV-19S_4:** I am afraid of losing my life because of Coronavirus-19

**FCV-19S_5:** When watching news and stories about Coronavirus-19 on social media, I become nervous or anxious.

**FCV-19S_6:** I cannot sleep because I’m worrying about getting Coronavirus-19

**FCV-19S_7:** My heart races or palpitates when I think about getting Coronavirus-19

***Patient health Questionnaire (PHQ-9) Scale***

**Answer to the following statements regarding your current status (i.e., last two weeks) using a four-item Likert-type scale.**

**Answers included: 1. Not at all 2. Several days 3. More than half the days 4. Nearly every day**

**PHQ-9_1:** Little interest or pleasure in doing things

**PHQ-9_2:** Feeling down, depressed or hopeless

**PHQ-9_3:** Trouble falling or staying asleep, or sleeping too much

**PHQ-9_4:** Feeling tired or having little energy

**PHQ-9_5:** Poor appetite or overheating

**PHQ-9_6:** Feeling bad about yourself-or that you are a failure or have let yourself or your family down

**PHQ-9_7:** Trouble concentrating on things, such as reading the newspaper or watching television

**PHQ-9_8:** Moving or speaking so slowly that other people could have noticed. Or the opposite-being so fidgety or restless that you have been moving around a lot more than usual

**PHQ-9_9:** Thoughts that you would be better off dead, or of hurting yourself

***Suicide-related questions (SRQs)***

**SRQ: Do you think about committing suicide, and are these thoughts persistent and related to COVID-19 issues?**

1. **Yes**
2. **No**

***Insomnia Severity Index (ISI)***

**For the first three statements (ISI_1 to ISI_3), please rate the current (i.e., last two weeks) severity of your insomnia problem(s) using a 5-point Likert scale: 1. None 2. Mild 3. Moderate 4. Severe 5. Very severe**

**ISI_1:** Difficulty falling asleep

**ISI_2:** Difficulty staying asleep

**ISI_3:** Problems waking up too early

**ISI_4: How SATISFIED/DISSATISFIED are you with your CURRENT sleep pattern?**

1. Very satisfied
2. Satisfied
3. Moderately satisfied
4. Dissatisfied
5. Very dissatisfied

**ISI_5:** **How NOTICEABLE to others do you think your sleep problem is in terms of impairing the quality of your life?**

1. Not at all noticeable
2. A little
3. Somewhat
4. Much
5. Very much noticeable

**ISI_6: How WORRIED/DISTRESSED are you about your current sleep problem?**

1. Not at all Worried
2. A little
3. Somewhat
4. Much
5. Very much worried

**ISI_7:** **To what extent do you consider your sleep problem to INTERFERE with your daily functioning (e.g. daytime fatigue, mood, ability to function at work/daily chores, concentration, memory, mood, etc.) CURRENTLY?**

1. Not at all interfering
2. A little
3. Somewhat
4. Much
5. Very much interfering
